# Effect of laser beam incidence angle on weld formation mechanism and corrosion resistance of T2 copper/304 stainless steel

**DOI:** 10.1038/s41598-024-57316-3

**Published:** 2024-03-21

**Authors:** Yubo Wang, Wei Liu, Heng Cui, Guipeng Lu

**Affiliations:** 1 Key Laboratory of Advanced Structural Materials, Ministry of Education and School of Materials Science and Engineering, Changchun University of Technology, Changchun, 130012 People’s Republic of China; 2 CCRC Changchun Railway Vehicles Co., Ltd, Changchun, 130062 People’s Republic of China; 3 FAW Tooling Die Manufacturing Co., Ltd, Changchun, 130013 People’s Republic of China

**Keywords:** Laser brazing, Element diffusion, Forming mechanism, Corrosion resistance, Materials science, Structural materials, Techniques and instrumentation

## Abstract

Over the last 20 years, industry interest in copper has increased. Its application in the petrochemical, automotive, and nuclear power industries highlights the need for new research directions especially in the joining of copper to other metals. In this work, lap joint of 304 stainless steel and T2 copper precoated with Cu–Mn–Ni filler metal was performed by laser brazing. The aim of this study is to characterize the influence of laser beam incidence angle on the welded joint forming mode, microstructure, elements diffusion and corrosion resistance. According to the findings, the joint is classified as a welded joint when the laser beam incidence angle is 80°, and as a welded-brazed joint when it’s 90°, 70°, or 60°. The microstructure is mainly composed of Cu-rich and Fe-rich phases, Mn in the Cu-rich phase aggregation and Cr in the Fe-rich phase aggregation. In the fusion zone (FZ) the content of less than 50% of the liquid will be in the form of supersaturated droplets in the matrix of the other side. The local corrosion pair that the copper steel matrix and liquid drop produce in the FZ speeds up the dissolution of the Cu-rich phase, which effected corrosion resistance of the joint.

## Introduction

Copper-steel welded structure not only can combine the excellent electrical and thermal conductivity of copper, but also can play a stainless steel corrosion resistance and greater mechanical properties, this welded structure can meet the structure engineering of the material qualities of different purposes. However, there are issues with welding copper-steel in the industry. Due to variations in their physical and chemical properties, the base material of stainless steel is vulnerable to copper penetration cracks during the welding process, and the welding seam is susceptible to cracks. The heat-affected zone on the copper side affects the mechanical properties of the joints due to excessive grain growth, which restricts the technical application of the copper-steel composite components^1–3^.

In order to prevent metal from melting, some researchers attempt to weld dissimilar metals, using solid state welding techniques including friction stir welding^4,5^ and explosive welding^6,7^. However, friction stir welding is primarily employed for softer metals, including aluminum and magnesium alloys. High-strength metals, such as copper alloys and steel, require more expensive tool materials and more exact tool incidence setting, therefore the cost is higher. More rigorous safety assessments and exceptionally safe experimental conditions are necessary for explosive welding. Some researchers have tried to use electron beam welding (EBW)^8–10^ and metal inert-gas welding (MIG) welding^11^ to connect copper-stainless steel dissimilar materials. It is found that electron beam welding is susceptible to interference from electromagnetic environment such as ferromagnetism in steel, making it difficult to accurately control the heating position, and another major drawback of EBW is the necessity of vacuum condition. After MIG welding, softening occurs in the heat-affected zone (HAZ) on the copper side and significant reduction in mechanical properties of joint. Laser welding has the advantages of accurate control of heat input, high energy density, narrow HAZ after welding and non vacuum conditions, at the same time the joint can be modified by using filler metal. Thus, the study on laser welding of dissimilar metals has many applications in engineering.

Ramachandran et al.^12^ studied the effect of laser welding process on the microstructure and mechanical properties of copper-stainless steel joint. Through careful study, it was found that there was obvious dendritic structure at the weld interface of stainless steel side, the weld interface of copper side had obvious grain coarsening region. The average microhardness of the FZ was (216 ± 22) HV, and the ductile failure happened at the weld interface of copper side. Suga et al^13^. studied the effect of brazing conditions and filler metal composition on the microstructure and strength of copper-steel lap joint by laser brazing, it was discovered that the tensile-shear was correlated with the bonding width of the copper based materials, whereby the Ni–Cu type brazing material provides comparatively high welding speed and joint strength. Mannucci et al.^14^ performed continuous laser welding of Cu/316 L stainless steel and investigated the effects of laser power, welding speed and beam incidence on microstructure and tensile properties. Excessively welding speed and laser power lead to the formation of hot cracks in the joint, and the microstructure of the joint depends on the copper content and determines the corrosion resistance of the joint.

The present investigation primarily focuses on the microstructure, mechanical properties and process window enhancement of copper-steel laser welding. The element distribution at the weld interface of dissimilar materials have a significant impact on the performance of joints, but there are relatively few studies on these topics. Therefore, it is important to investigate the diffusion of interfacial elements and the structure of the interfacial layers. In this study, the method of precoating brazing material is used to realize the laser welding of T2 copper and 304 stainless steel. The microstructure, element distribution, forming mechanism and corrosion resistance of the laser brazed joint of T2 copper and 304 stainless steel dissimilar metals are analyzed and investigated in detail.

## Materials and methods

**Figure 1:**
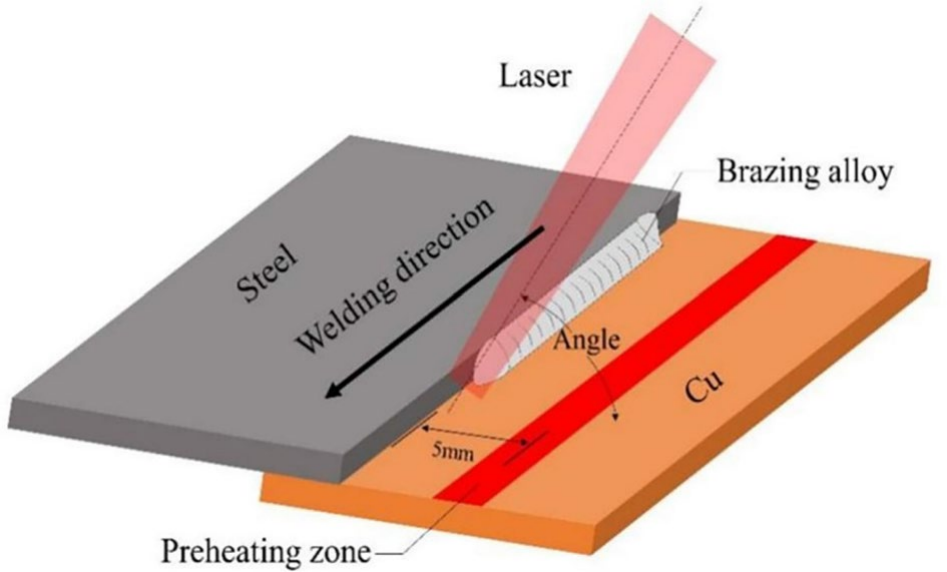
Welding assembly drawing.

The experimental materials are 150 mm × 80 mm × 1 mm 304 austenitic stainless steel sheet and T2 copper sheet, the filler metal is a copper-based (Cu–40Mn–20Ni, wt%) powder (melting point range 950–960 ℃, brazing temperature range 1000–1050 ℃, particle size 150–300 μm), the base materials and brazing materials composition as shown in Table 1. Grinding of metal surfaces before welding, in order to remove the material surface of the oxide film and other impurities as well as to increase the roughness of the metal surface, grinding and cleaning of the metal surface with acetone, and the interface to be precoating with filler material for welding (thickness ≤ 5 mm). The JHM-1GXY-500B pulse laser welder is used to join the simples and the welding assembly is shown in Fig. 1. During laser process, laser power of 400 W, laser pulse width of 20 ms, pulse frequency of 5 Hz, defocusing distance of − 2.5 mm, welding speed of 90 mm/min, argon gas flow of 15 L/min, and laser beam incidence angles are set to be 60°, 70°, 80°, and 90° under these experimental conditions. As shown in Fig. 1, due to the high thermal conductivity of copper. The base material at a distance of 5 mm from the joint on the copper side is preheated before welding with the same defocusing distance, laser power and welding speed used for welding, and then the joint is welded immediately.

**Table 1:** Composition of T2 copper, 304 stainless steel and filler metal (mass fraction wt%).

T2 copper	Cu	Fe	Ni	S	Pb	Sn	Zn	Else
	99.90	0.005	0.005	0.005	0.005	0.002	0.005	0.1
304 stainless steel	Mn	Ni	Cr	Si	P	N	C	S
	2.0	8.0–10.5	18.0–20.0	1.0	0.035	0.1	0.08	0.03
Cu–Mn–Ni filler metal	Cu			Mn			Ni	
	40			40			20

After welding, 8 mm × 8 mm metallographic specimens were machined perpendicular to the direction of welding. After polishing, the joints are etched for 2 s with FeCl_3_ solution (FeCl_3_:HCl:H_2_O = 2:1:10). The macrostructure and microstructure of the joints are characterized by Leica DMI8 optical microscope. The microstructure and elemental distribution of the joints are investigated using a Zeiss Gemini Supra 40 field scanning electron microscope (SEM) combined with energy dispersive spectroscopy (EDS). The current impedance of the joints are measured by CHI604E electrochemical workstation. The non-test area is encapsulated in epoxy resins with an electrolyte of 3.5% NaCl solution. The experimental device adopts a typical three electrode system, with a saturated calomel electrode as the reference electrode and a platinum electrode as the auxiliary electrode, with an effective area of 1 mm × 2 mm, scanning range of − 0.5 to 0.8 V and scanning rate of 0.01 V/s.

## Results and discussion

### Microscopic morphology

**Figure 2:**
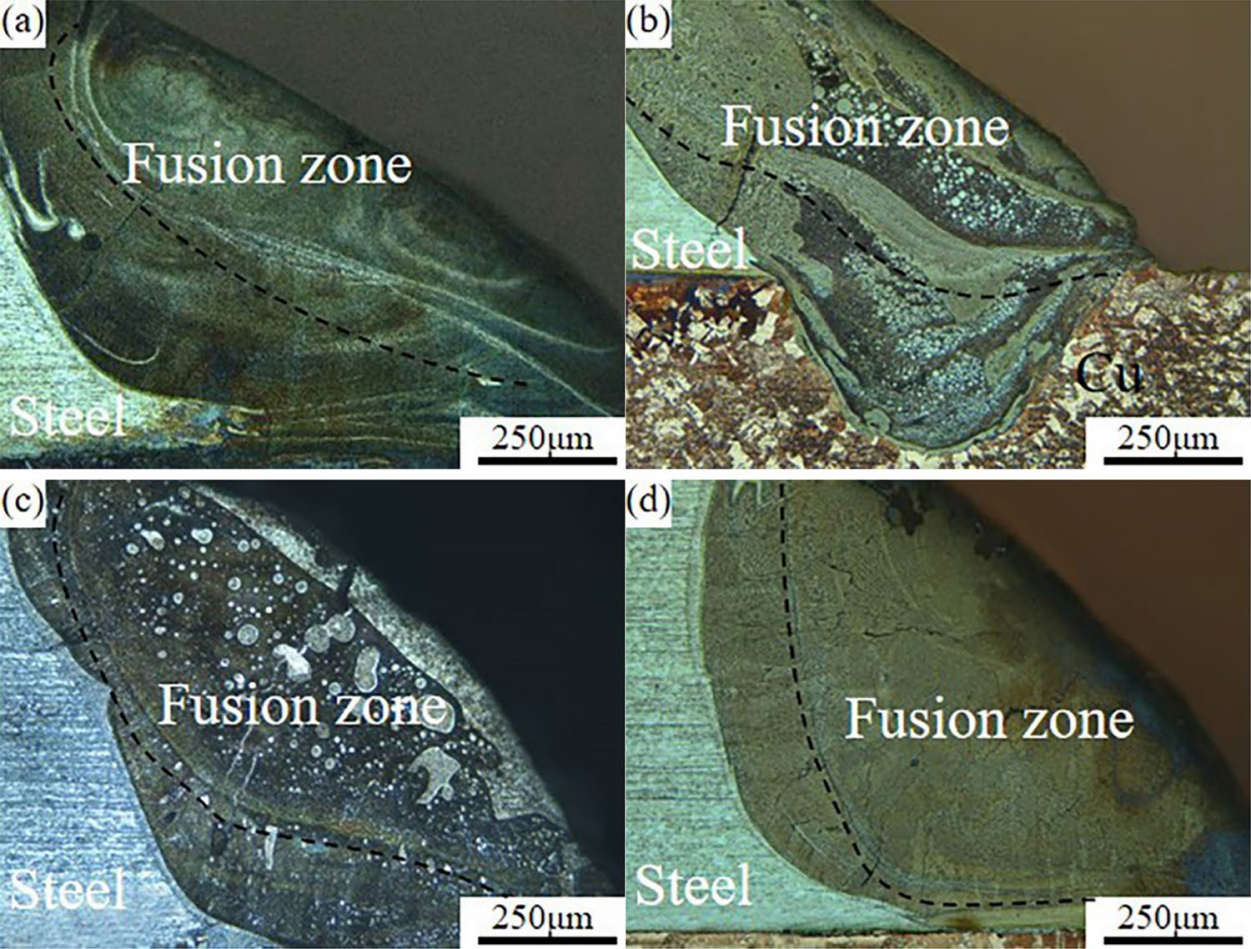
Welding micro-morphology obtained under dissimilar laser beam incidence conditions (**a**) 90° (**b**) 80° (**c**) 70° (**d**) 60°.

The cross-sectional morphology of the welded joint obtained under dissimilar laser beam incidence angles are shown in Fig. 2, and it is found that the laser welding of dissimilar alloys of copper-stainless steel has two different connection modes. One is welded joint formed by mixing and solidification the partially melted copper-steel base material with the brazing material at an incidence angle of 80° in Fig. 2b. The other is welded-brazed joint formed when the angles are 90°, 70° and 60° respectively, where only the brazing material and the stainless steel are melted by laser heating and the copper remains solid in Fig. 2a,c and d. It is evident that the angle at which the laser beam incidence occurs determines the weld type. The connection mode changes from welded-brazed to welded then to welded-brazed when laser beam incidence angles decrease. It is analyzed that when the angle is 90°, the laser beam is perpendicular to the joint, and most of the energy is reflected off by the copper, resulting in insufficient heat input for the weld to form the welded-brazed joint on the copper side. When the angle is 80°, the laser beam is no longer perpendicular to the copper base material, the reflected energy is greatly decreased, and the effective laser energy is greatly increased, resulting in partial melted of the copper and metallurgical reactions with the brazing material to form the welded joint. As the laser beam incidence angles continues to decrease to 70° and 60°, the laser beam is gradually shifted to the steel side, so that the steel side obtains more energy. Therefore, the energy absorbed by the copper side is gradually reduced to form the welded-brazed with brazing filler metal.

The brazing material and the base material differ in composition, which causes the joint to have noticeable layered structures. There is a “dividing line” in the FZ that divides its upper and lower regions in Fig. 2. As the laser beam incidence angles decrease, there is a corresponding angular change between the dividing line and the fusion line on the steel side. It can be found that with decreasing incidence angles the laser spot is gradually shifted to the steel side resulting in an increase in the amount of melting of the base material on the steel side. Since the laser acts regularly in pulses on the welded joint, the laser forms the circular “weld joint” at the first irradiation of the weld seam. As the laser moves, the second welded point builds on top of the first one (step size 0.1 ms), the weld seam is created by several welded points that are evenly stacked, and the “dividing line” is the junction of the two joints during the welding process that the dotted line in Fig. 2. Due to the time difference between the welding sequence and the cooling rate of the molten pool, the front welded point is affected by the temperature field of the back welded point, the grain junctions are heated twice to form the vertical joints growing columnar crystals. As a result, a “dividing line” is formed at the macro level. The Fig. 2 also illustrates how the crystallization morphology at the FZ at 90° and 60° differs from that at the FZ with laser beam incidence angles of 80° and 70°. And the upper part of the FZ has a considerable amount of spherical particles of varying sizes, which could be related to the action of the laser beam and the flow of the molten pool during laser welding. Since copper-stainless steel have no solid solubility in each other, it is inferred that the spherical particles in the FZ are Fe-rich phase particles after condensation by using EDS later in the SEM. It has been documented^15,16^ that the proportion of copper-steel content affects the microscopic morphology of welded joint and at different proportions copper-steel liquids will be present in different forms in the joint.

### The distribution of elements in the fusion zone

**Figure 3:**
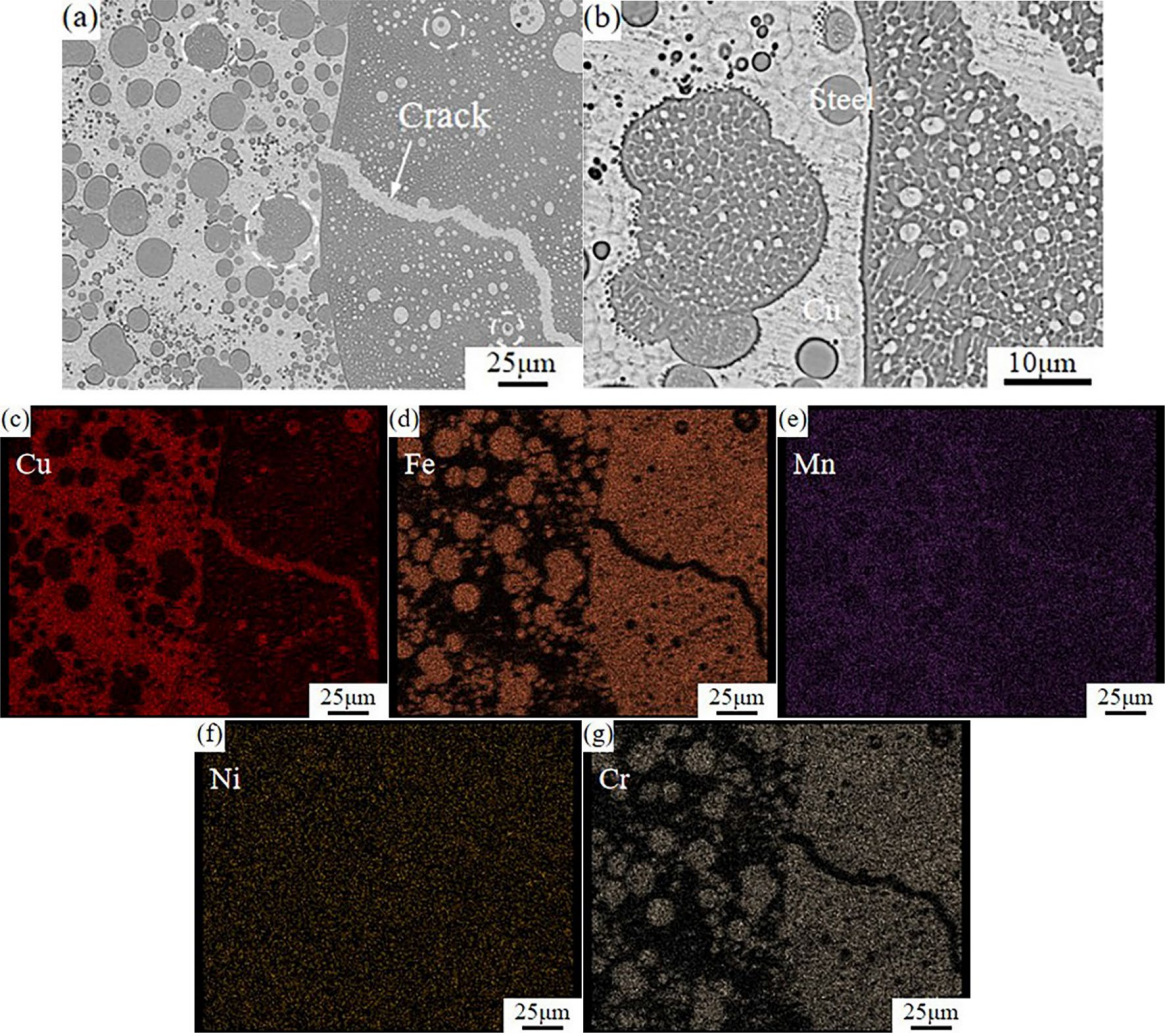
Elemental mapping of FZ (**a**) SEM image of the FZ (**b**) SEM image of the FZ (**c**) Cu (**d**) Fe (**e**) Mn (**f**) Ni (**g**) Cr.

Fig. 3 shows the SEM images of the FZ of the joint and the elemental distribution maps at a laser beam incidence angle of 70°, where the dark gray stainless steel matrix and the light gray Cu matrix. The Fe-rich particles and Cu-rich particles distributed within the FZ in Fig. 3. A significant amount of Fe-rich and Cu-rich particles define the microstructure in the FZ, as seen in Fig. 3a. On the left side of the figure, there is a homogeneous distribution of spherical Fe-rich particles in the Cu matrix, along with the fusion of Fe-rich particles with one another. The spherical Cu-rich particles on the right side of the figure are uniformly distributed in the steel matrix, where the diameter of the spherical Fe-rich phase is greater than that of the spherical Cu-rich phase. Because of the presence of Fe-rich dendrites on the surface of the Fe-rich particles that are growing toward the interior of the Cu matrix, the Fe-rich particles within the joint can serve as nucleating agent for heterogeneous nucleation during the cooling phase of laser brazing. Another characteristic of the FZ is the formation of micro-cracks in the stainless steel side of the welding process, the cracks close until invisible when cooled because liquid copper fills the cracks with a “self-healing” effect. Figure 3b shows an SEM image of the FZ, where it is observed that the Fe-rich particle composition is heterogeneous. The Fe-rich particles and the steel matrix exhibit the reticulated distribution of secondary precipitates of the Cu phase, with smaller Fe-rich particles found in the Cu-rich particles as well.

Welding of metals is different due to their elemental composition and physicochemical properties, and the study of elemental diffusion and distribution at the weld interface can better improve the quality of welded joint through process control. In order to investigate the compositional distribution of the laser brazed joint of T2 copper and 304 stainless steel, the FZ of the joint in Fig. 3a is analyzed by the element distribution maps, and the results are shown in Fig. 3c–g. In Fig. 3, it can be seen that the Cu and Fe do not undergo significant fusion because of copper-steel do not form the intermetallic compounds, but exist in each other's boundaries as solid solutions. The Ni is uniformly distributed throughout the FZ and no significant enrichment occurs. One the one hand, Ni and Cu can exist in the endless solid solutions and enhance the Cu matrix in some degree. Ni in the brazing material can also be firmly dissolved in the metal to enhance the stainless steel's qualities. The Ni in the brazing material is uniformly distributed in the FZ, because copper-steel are the primary metals used in welding. While Mn is not as uniformly distributed in the FZ as Ni is, it is evident that the Cu matrix has a greater Mn content than the steel matrix, because the Cu side is brighter in color. This is primarily because the brazing material has a much greater Mn content than the stainless steel itself, and under laser radiation and heating conditions, Mn will be enriched in the Cu-rich phase^17,18^. As a result, the Cu-rich particles and Cu matrix in the FZ contain a significantly greater amount of Mn than the Fe-rich particles and Fe matrix. It is evident that Cr–Cu solid solution can not form under the fast cooling circumstances of laser welding, since Cr is practically insoluble below 600° and reaches its maximal solid solubility in Cu at the eutectic temperature of 1076°. Furthermore, Cr is exclusively found in base materials and Fe-rich particles in the elemental mapping results, because it is pro-Fe and form Fe solid solution.

**Figure 4:**
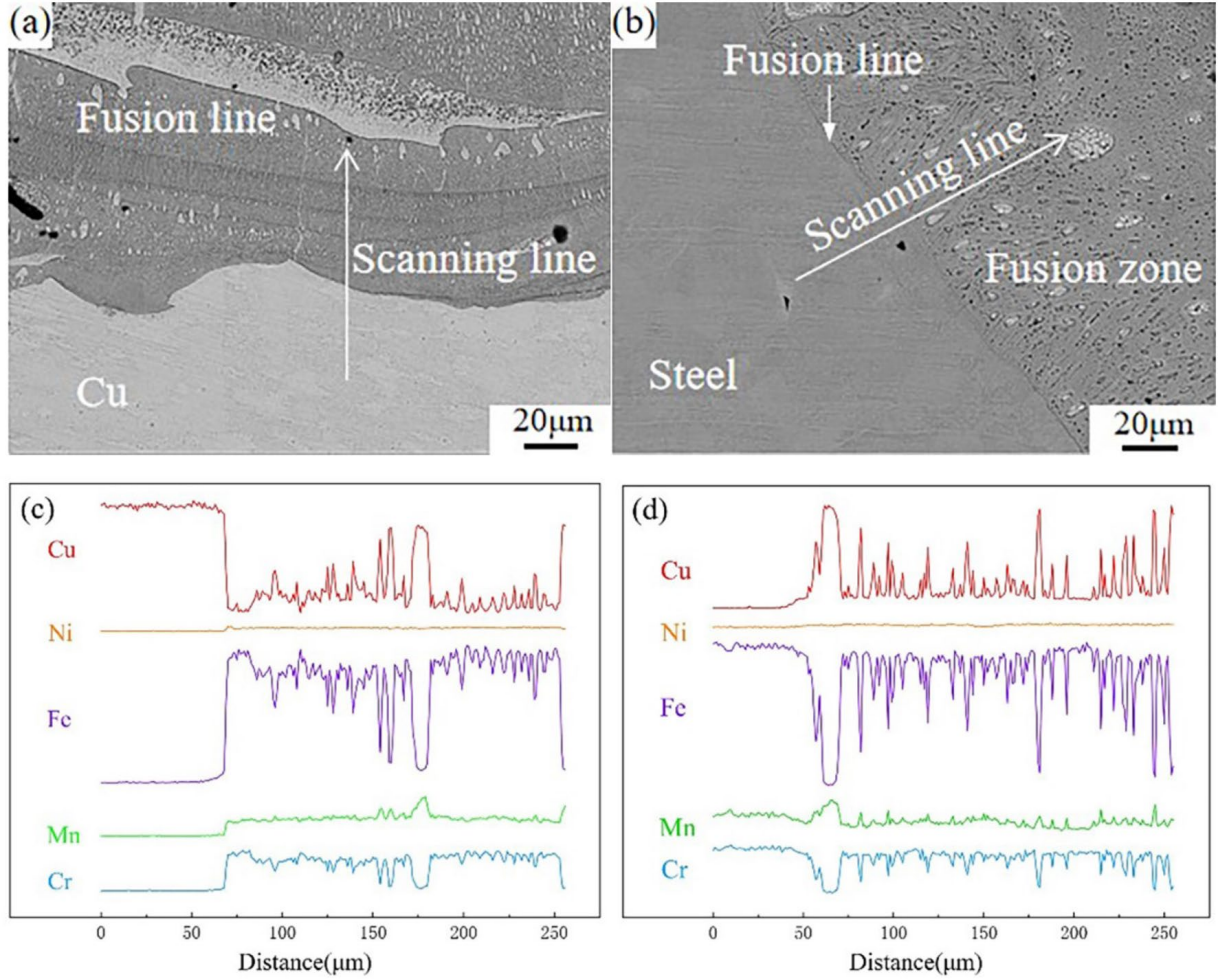
(**a**) Copper side electron backscatter diffraction (**b**) Steel side electron backscatter diffraction (**c**) Copper side line scanning (**d**) Steel side line scanning.

The SEM images of the copper-stainless steel joint interfaces are displayed in Fig. 4a and b. In order to investigate element diffusion in the interface region between the FZ and the two base materials, EDS line scanning analysis of the joint interface is performed respectively. In Fig. 4c and d, the content of Fe and Cu elements has an opposite distribution, while the content of Cu elements increases and the content of Fe elements decreases. This is because copper-stainless steel can not form solid solution and repel in a limited area of the FZ. This finding is compatible with the elemental mapping results, showing that Mn diffuses and collects into the Cu-rich phase under the operation of the laser beam along with the elevation of Cu. Meanwhile, when the Fe content decreases, the Cr solid solution in the Fe-rich particles also decreases, and only the Ni is uniformly distributed in the stainless steel base material and the FZ. In Fig. 4c, the transition layer of each element at the interface connection between the FZ and the copper base material is very narrow, and the content of Ni element is significantly increased, indicating that Ni element was enriched at the transition layer (Distance 50–75 μm). This suggests the transition layer at the interface between the stainless steel and the fusion zone is relatively wide, and the concentration of each element in the transition layer does not change significantly. Figure 4d shows the diffusion of the Cu into the transition layer during the welding process. The Mn content in the transition layer increases along with the Cu content after decreasing (Distance 25–75μm) and becoming consistent with the distribution of the Cu in Fig. 4d. It is analyzed that during the cooling process, the generation of Cu-rich phase makes the Mn continuously gather to the Cu-rich phase resulting in the Mn-poor transition layer. And the temperature at the connecting interface decreases faster and the drive force of element diffusion is low, resulting in this layer being less susceptible to element diffusion.

### Liquid phase separation

**Figure 5:**
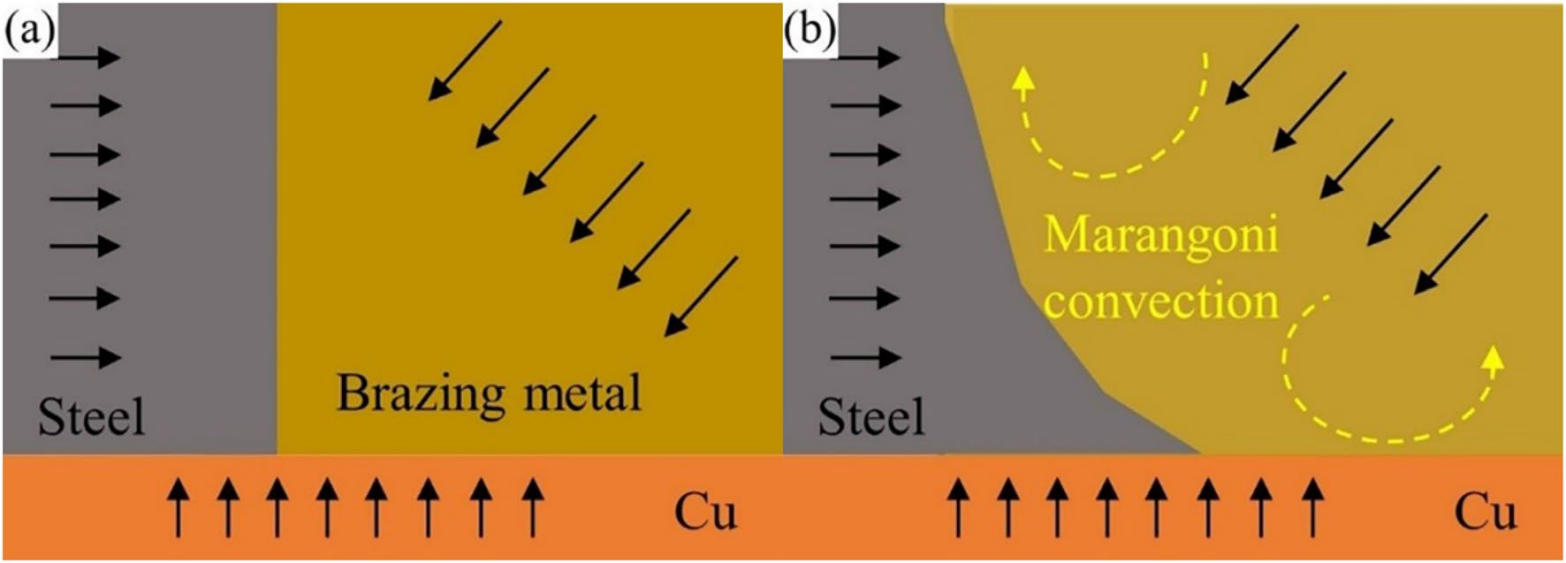
(**a**) Schematic diagram of welding joint forming (**b**) Marangoni convection.

In the initial stage of laser brazing, the laser beam is applied to the brazing metal and copper-stainless steel base material. As seen in Fig. 5a, the molten metal is now subject to three forces: the rigid binding force between the copper-stainless steel base materials, the reaction force caused by the metal melting and evaporating under the laser beam^19^. At the same time, the brazing metal and part of the stainless steel melt and mix form the molten pool. In the molten pool, the Marangoni effect is triggered because of the difference in liquid surface tension between copper-stainless steel^20^, and Marangoni effect does not with the variation of incidence angle. The surface tension is influenced by temperature for pure metals and most alloys, the greater the temperature, the lower the surface tension; the edge of the molten pool is next to the foundation material made of copper-stainless steel, the higher temperatures to occur in the middle of the weld pool at the laser beam incidence zone. As a result, the liquid metal near the molten pool border has the greater surface tension than the liquid metal in the pool middle. The metal in the middle of the molten pool will flow to the edge due to the action of this surface tension gradient, and the liquid metal will reflux back to the middle and upper part of the molten pool under the strict constraints of the copper-stainless steel base materials. This flow model is illustrated in Fig. 5b. The liquid metal in the middle-upper part of the molten pool in turbulent state eventually cools and crystallizes to form a weld, the process that can be clearly seen in the microstructure of the joint in Fig. 2c.

Since the laser pulse was used in this investigation, the joint receives regular and intermittent laser beam action. The joint molten pool is fully self-cooling during the period between two laser actions, it no longer gets laser beam activity and no heat input. When the laser beam acts on the joint, brazing metal and part of the stainless steel base material is heated to become liquid. High temperature copper-stainless steel solubility increases, making the chemical potential gradient increases, resulting in copper-stainless steel elements occurring in the mutual diffusion, and at high temperatures in each other solid solution to each other's matrix, the formation of metallurgical bonding. Under subcooling, the molten pool moves into the miscibility gap of the Cu–Fe alloy^21^, where the Cu–Fe liquid phases separate out of the liquid metal.

**Figure 6:**
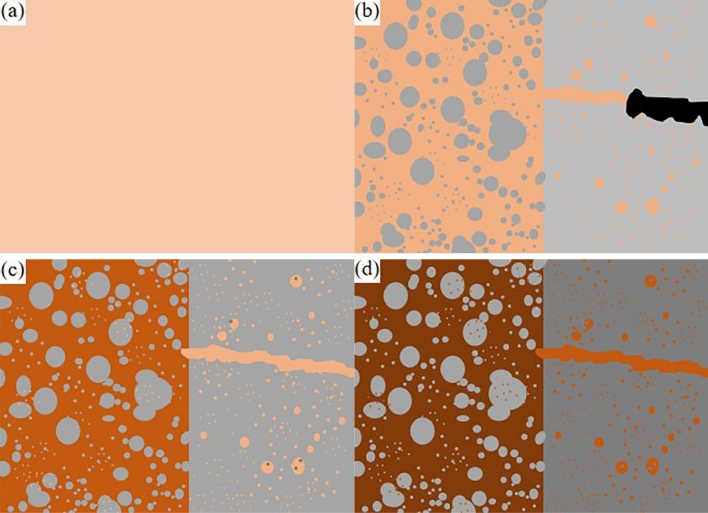
(**a**) Schematic diagram of Cu–Fe liquid phase separation (**b**) Primary separation (**c**) Secondary separation (**d**) Complete the liquid phase separation process.

From the kinetic and thermodynamic point of view, the refractory fusion system of Cu–Fe alloys has a very strong tendency to liquid phase separation. Nakagawa et al^22^. found that liquid phase separation occurs in Cu–Fe alloys under supercooling conditions. As shown in Fig. 6a–d, the molten pool metal is fully mixed under the impact of the laser beam. During the cooling process, the Fe-rich phase is the first to be cooled and molded, and the Cu-rich phase which in the Fe-rich liquid phase precipitates out and is distributed in the Fe-rich phase at the same time. At this point, the Cu-rich phase in the liquid state will fill the gap between the Fe-rich phase, with the further cooling of the molten pool, Cu and Fe atoms in the Fe-rich phase and Cu-rich phase of the solid solubility is further reduced and precipitated in the form of particles to complete the liquid phase separation process. Chen et al. proposed several factors affecting liquid-phase separation^23^, including the cooling rate, the extent of the miscibility gap and the diffusion rate of the metal atoms. From the Cu–Fe binary phase diagram, the temperature range of the miscibility gap is relatively small, close to the line of liquid phase of Cu–Fe, due to the characteristics of the weld seam cooling fast and the high thermal conductivity of Cu so that the molten pool can be rapidly subcooled to meet the subcooling conditions. In addition, the rate of atomic diffusion decreases with decreasing temperature, and the already separated liquid phases do not fuse again. It can be seen that the laser brazing of T2 copper and 304 stainless steel satisfies the condition of liquid phase separation, so the Cu–Fe liquid phase separation is widely existed in the FZ of the joint under the action of strong convection of the laser beam.

If the liquid copper content in the molten pool is greater than the liquid stainless steel content, the particles separated in the lateral liquid phase come from the stainless steel. If the liquid copper content is higher than the liquid stainless steel content in the molten pool, the particles separated in the lateral liquid phase come from the stainless steel. This explains well the microstructure of Fig. 3a. From the phase diagram of the Cu–Fe binary alloy, it can be seen that the miscibility gap △T of the Fe-rich phase is greater than the miscibility gap △T of the Cu-rich phase, and then combined with the classical nucleation theory^24^:

$$\Delta G=(16\uppi \sigma^\wedge3 Tm^\wedge2)/(3(Lm\cdot \Delta T)^2 )$$

Among others: △G is the critical nucleation work, σ is the surface energy per unit area of crystal, Tm is the liquidus temperature of metal, Lm is the latent heat of crystallization, △T is the undercooling of melt. Since parameters such as σ are the same, △T is negatively correlated. It can be concluded that the critical nucleation work (△G) of the Fe-rich liquid phase is smaller than that of the Cu-rich liquid phase, which is easier to nucleate during the cooling process of the molten pool. Combined with the heat dissipation characteristics of the welded joint, the joint will first nucleate in the Fe-rich liquid phase at the interface between the molten pool and the copper base material. As the temperature decreases, the Cu-rich particles in the Fe-rich liquid phase precipitate out and distribute in the intergranular gap. The supercooling degree at the interface between the molten pool and the copper base material is greater than that inside the molten pool, so the precipitated Cu-rich phase particles are smaller in size, and this phenomenon is consistent with the EDS at the interface in Fig. 4a. The Cu-rich liquid phase has a small range of sub-stable miscibility gaps and solidification occurs after the Fe-rich liquid phase. The cooling rate inside the molten pool is slower than that at the Cu side interface, so the Fe-rich liquid-phase nucleation grow longer, and this phenomenon can be clearly observed in Fig. 2c.

As the joint temperature gradually decreases, the Fe-rich particles inside the molten pool reach the miscibility gap, the solid solubility of Cu atoms in the Fe-rich particles decreases and precipitates out in the Fe-rich particles, and the secondary liquid-phase separation of the Cu–Fe liquid phase occurs, and the final liquid-phase separation of the joints formed by the microstructure and morphology of the joints are shown in Fig. 3a and b.

### Corrosion resistance

**Figure 7:**
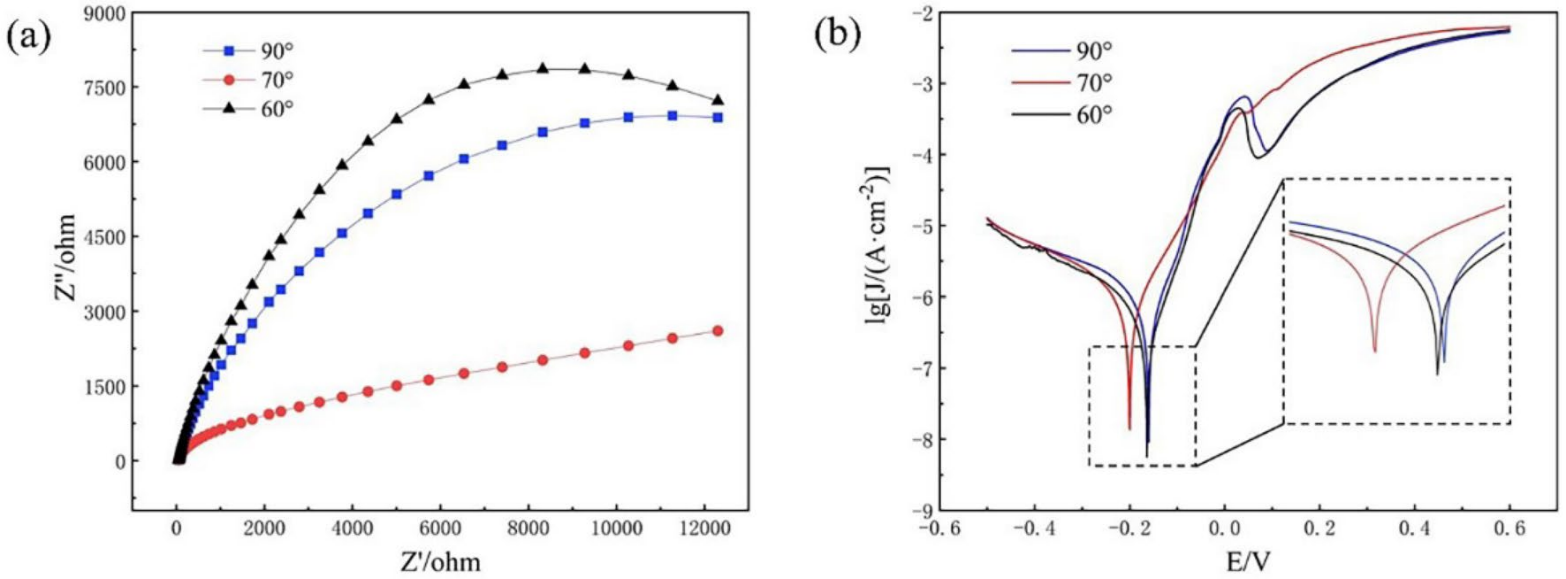
(**a**) Impedance curves (**b**) Polarization curves.

In order to investigate the corrosion resistance of the welded joints, three sets of specimens in welded-brazed mode are selected to test the corrosion resistance of the joints in a NaCl solution with a mass fraction of 3.5%. The polarization curves and impedance curves of the three sets of specimens are shown in Fig. 7a and b. It can be seen that the radius of arc in impedance of the specimen with the angle of 60° is the largest, indicating that its polarization resistance is the largest and most corrosion-resistant. Conversely, the specimen exhibiting the lowest the radius of arc in impedance at 70° signifies the smallest effective corrosion resistance. According to the size of the impedance arc radius of the impedance curves, the order of corrosion resistance of welded joints with dissimilar laser beam incidence angles are 60° > 90° > 70°. Figure 7b shows the polarization curves of the three sets of specimens, and the results obtained by fitting the polarization curves are shown in Table 2. The corrosion current density I_corr_ characterizes the corrosion rate of the material^25^, the smaller the current density the more difficult the charge transfer during corrosion, indicating that the material is less susceptible to corrosion. As can be seen from Table 2, the corrosion resistance of the three sets of specimens are in the order of 60° > 90° > 70°, and this result is also consistent with impedance curves results.

**Table 2:** Electrochemical parameters simulated from the polarization curves of dissimilar laser beam incidence angles.

Laser beam incidence angle (°)	E_corr_ (V)	I_corr_ (A·cm^-2^)
90	-0.16	6.652
70	-0.20	7.787
60	-0.164	3.806

**Figure 8:**
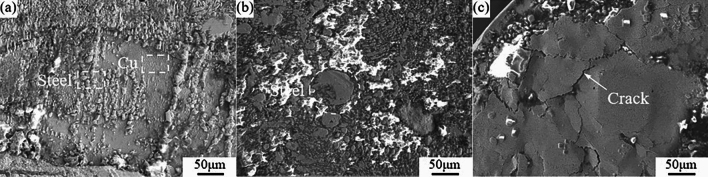
Corrosion morphology of joint of dissimilar laser beam incidence angles (**a**) Copper and stainless steel in the joint (**b**) Stainless steel in the joint (**c**) Crack.

Direct contact between the two materials, different corrosion potentials, and continuous electrolyte in contact are necessary for galvanic corrosion of coupling to occur. Low potential materials are etched faster due to the galvanic corrosion, which ultimately compromises the material performance and lifespan. Figure 8 shows the morphology of the joint after interfacial corrosion at dissimilar laser beam incidence angles, from which it can be clearly seen that the interfacial morphologies are different for dissimilar stainless steel contents in the joint. In Fig. 8a, the whole joint is based on copper and the steel is dispersed in the FZ with Fe-rich particles. During the corrosion process, the copper is gradually etched leaving Fe-rich particles and forming the morphologies. Figure 8b shows the closer approach to 1:1 in the Fe–Cu liquid phase proportion, and the increasingly clear liquid phase separation. The state of solidification of the metal is changed, and the Fe-rich phase particles gradually become greater and are distributed in the FZ. When the Fe-rich phase particles contain higher concentrations of Cr and Ni, they will form the dense oxide film that will impede the corrosion process, resulting in minimal changes to the surface morphology of the particles. Steel progressively outnumbers Cu in the FZ as the laser beam incidence angle decreases, the FZ with steel serving as the substrate has Cu-rich phase in the form of granular and fill cracks. Cu particles and fill cracks are etched, pitting and strip corrosion pits are visible on the surface.

In summary, the increase of stainless steel content in the FZ has small effect on the corrosion resistance of the joints, but the structure formed by the liquid phase separation of copper-steel in the FZ during the cooling process has the great influence on the corrosion resistance of the joints.

## Conclusions

In this work, The laser beam incidence angle was used to achieve laser welding of T2 copper and 304 stainless steel. The laser brazed joint of T2 copper and 304 stainless steel dissimilar metal were analyzed and studied, the main conclusions are as follows:

1. Laser brazed joints with dissimilar laser beam incidence angles are categorized into “welded mode” and “welded-brazed mode”. As the laser beam incidence angle decreases, the stainless steel base material involved in the welding process increases and the proportion of copper-steel content in the joint changes, which affects the microstructure of the joint.
2. The FZ of the joint consists mainly of the Cu-rich phase and the Fe-rich phase. The Cu-rich phase and the Fe-rich phase can not form solid solutions and repel each other. Mn in the FZ accumulates in the Cu-rich phase under laser radiation and heating conditions, resulting in the Mn-poor interface zone between the Fe-rich phase and the stainless steel. The Ni is uniformly distributed throughout the FZ and can form solid solution with Cu–Fe.
3. Liquid phase separation occurs between the brazing filler metal and the partially melted stainless steel base material, with the more being used as a matrix and the less being present in the matrix as Cu-rich or Fe-rich phase particles. At the later stage of molten pool cooling, Cu–Fe atoms in the Cu-rich and Fe-rich liquid phase precipitate out due to the decrease of temperature, and the secondary liquid phase separation occurs.
4. The increase of stainless steel content in the joint can increase the corrosion resistance of the joint to the certain extent, and the microstructure of the joint has the greater impact on the corrosion resistance of the joint. The separation of the Cu–Fe liquid phase leads to the appearance of the primary battery with steel as cathode and copper as anode during the corrosion process, resulting in the form of galvanic corrosion, which accelerates the dissolution of the Cu-rich phase and the form of the large amount of corrosion pits under the action of the galvanic corrosion.

## Data Availability

The datasets used and analysed during the current study available from the corresponding author on reasonable request.
